# Spatio-Temporal Variation in the Prevalence of Major Mastitis Pathogens Isolated From Bovine Milk Samples Between 2008 and 2017 in Ontario, Canada

**DOI:** 10.3389/fvets.2021.742696

**Published:** 2021-11-03

**Authors:** Kamal Raj Acharya, Gabrielle Brankston, Durda Slavic, Amy L. Greer

**Affiliations:** ^1^Department of Population Medicine, University of Guelph, Guelph, ON, Canada; ^2^Animal Health Laboratory, University of Guelph, Guelph, ON, Canada

**Keywords:** spatio-temporal variation, prevalence, mastitis pathogens, Canada, bovine

## Abstract

An understanding of the spatio-temporal distribution of several groups of mastitis pathogens can help to inform programs for the successful control and management of mastitis. However, in the absence of an active surveillance program such information is not readily available. In this retrospective study we analyzed passive surveillance data from a diagnostic laboratory with an aim to describe the spatio-temporal trend of major mastitis pathogens between 2008 and 2017 in Ontario dairy cattle. Data for all milk culture samples submitted to the Animal Health Laboratory (AHL) at the University of Guelph between 2008 and 2017 was accessed. Descriptive analyses were conducted to identify the major pathogens and Chi-square goodness-of-fit tests were used to compare between multiple proportions. Likewise, univariable logistic regression analysis was performed to determine if there was a change in the probability of isolating the major mastitis pathogens depending on geography or time. Seasonality was assessed by calculating the seasonal relative risk (RR). Of a total of 85,979 milk samples examined, more than half of the samples (61.07%) showed no growth and the proportion of samples that showed no growth almost halved during the study period. Of the samples (36.21%, *n* = 31,133) that showed any growth, the major bacterial pathogens were *Staphylococcus aureus* (15.60%), Non-aureus Staphylococci (NAS) (5.04%), *Corynebacterium* spp. (2.96%), and *Escherichia coli* (2.00%). Of the NAS, the major species reported were *Staphylococcus chromogenes* (69.02%), *Staphylococcus simulans* (14.45%), *Staphylococcus epidermidis* (12.99%), and *Staphylococcus hyicus* (2.13%). A temporal change in the prevalence of contagious pathogens like *S. aureus* and *Corynebacterium* spp. was observed with an increasing odds of 1.06 and 1.62, respectively. Likewise, except for *Trueperella pyogenes*, the prevalence of all the major environmental mastitis pathogens increased during the study period. The isolation of most of the pathogens peaked in summer, except for *S. aureus, T. pyogenes*, and *Streptococcus dysgalactiae* which peaked in spring months. Interestingly, a regional pattern of isolation of some bacterial pathogens within Ontario was also observed. This study showed a marked spatio-temporal change in the prevalence of major mastitis pathogens and suggests that a regional and seasonal approach to mastitis control could be of value.

## Introduction

Mastitis, the inflammation of the mammary gland, is one of the most important production limiting diseases in dairy cattle (1). Farmers have prioritized mastitis as the second most important dairy cattle disease based on the National Dairy Survey in Canada (2). Although the case fatality rate for mastitis is low, it adversely affects farm profitability and can result in significant economic losses (1, 3). Costs due to both clinical and sub-clinical forms of the disease include production costs, treatment costs, and prevention costs (3–5). In addition, there are indirect costs to farmers due to the penalty imposed on milk with a high somatic cell count (6). While the relative importance of the cost components may vary according to the clinical forms of the disease, losses associated with both clinical and sub-clinical forms of mastitis can amount to an average of Canadian $662 per milking animal per annum for Canadian dairy farmers (5). Mastitis is also important from an animal welfare point of view as clinical mastitis causes pain in the animals (7).

The relationships between pathogen, animal, and farm environment and management factors play an important role in the causation, and hence management and treatment of mastitis (8, 9). The management of mastitis is particularly challenging as mastitis in dairy cattle is caused by several groups of pathogens like the most frequently isolated *Staphylococcus aureus*, and opportunistic pathogens like *Streptococcus* species, *Escherichia coli, Klebsiella* species, and coagulase-negative Staphylococci (3, 9). The relative frequency of isolation of these pathogens can vary temporally or spatially and can also depend on the production stage of the animal and the clinical form of the disease (2, 10–15). The treatment of mastitis in cows involves the use of antimicrobials via intramammary and/or parenteral routes, which has been associated with antimicrobial resistance of the mastitis pathogens and future treatment failure (16). This is especially important when antibiotics are used in dairy cows as blanket dry cow therapy without the identification of the causative pathogen and its antimicrobial sensitivity profile (17–20). Antimicrobial resistance (if developed against antibiotics that are critically important for human use) will be detrimental to public health as some of the mastitis pathogens are also human pathogens, opportunistic human pathogens, or can transmit antimicrobial resistance genes to human pathogens. Therefore, information on the pathogens isolated from animals with mastitis will (1) improve situational awareness related to mastitis pathogens in the Ontario dairy population, (2) support the design of mastitis control plans, and (3) help clinicians to identify the hazards that are present in the Ontario dairy environment.

While many cross sectional studies have documented the most common mastitis causing pathogens in Ontario (2, 10–15), understanding how the prevalence of these pathogens has changed over time is an important area of focus. In this study, we estimate the prevalence of mastitis pathogens and describe the overall spatio-temporal trend for the major mastitis pathogens between 2008 and 2017 in Ontario dairy cattle.

## Methods

This retrospective study examined laboratory data for all milk culture samples submitted to the Animal Health Laboratory (AHL) at the University of Guelph between 2008 and 2017. These milk samples comprise samples that were routinely submitted to the laboratory for only culture or culture and susceptibility testing. Samples were submitted to the AHL by veterinarians as a part of mastitis testing and are expected to represent a sample from a quarter of an animal udder, while some of the samples were composite samples. Additional information on the clinical stage of the disease, and/or treatment history of the animal was not available.

### Bacterial Isolation

At the AHL, a standard microbiological isolation technique was followed to isolate bacteria from a milk sample. Briefly, 10 μl of milk were inoculated on Columbia blood agar (BA) and, if <20 milk samples were submitted, also on MacConkey (MAC) agar. Blood agar plates were incubated at 35°C in the presence of 5% CO_2_ whereas MAC agar plates were incubated at ambient air at 35°C. All plates were checked for the presence of bacterial growth after 24 and 48 h of incubation. In addition, all milk samples were incubated at 35°C aerobically up to 5 days if no bacterial growth was detected on the initial culture. After 5 days of incubation the milk samples were re-plated on BA only and incubated and checked for the presence of bacterial growth as specified above. Before 2011 bacterial identification was done biochemically following standard operating procedures at AHL whereas from 2011 until 2017 bacterial identification was achieved using matrix assisted time-of-flight mass spectroscopy (MALDI-TOF MS). Briefly, individual bacterial colonies were smeared on stainless steel plate and covered by matrix [α-cyano-4-hydroxycinnamic acid (HCCA)]. The results were read using the Bruker MALDI Biotyper. All major mastitis pathogens were reported. If no bacterial growth was detected the results were reported as no bacterial growth. If bacterial growth was detected but there were no major mastitis pathogens present the results were reported as no bacterial pathogens. Overgrowth with mold and/or *Proteus* spp. and numerous bacterial species was reported as overgrowth with contaminants.

### Data Management and Statistical Analysis

The milk culture data obtained from AHL was tabulated using a spreadsheet (Microsoft® Excel® for Office 365). A single entry represents a test performed on a milk sample from a single mammary quarter, or composite milk from all or some quarters of an animal. Duplicate entries were removed. It was expected that the milk samples tested at the AHL would be representative of milk samples tested for mastitis in the province of Ontario, Canada as the Ontario Ministry of Agriculture, Food, and Rural Affairs (OMAFRA) subsidizes the laboratory costs for samples submitted to the AHL. Hence, only milk samples submitted from farms with an Ontario address were retained in the dataset. In this study we retained the test results corresponding to general bacteriological examination of the milk samples.

For qualitative analysis, pathogens that constituted at least 1.5% of the total isolates were selected for further analysis except for *Streptococcus dysgalactiae* which was included because of its known importance as a mastitis pathogen. This included bacteria representing contagious mastitis pathogens (*S. aureus* and *Corynebacterium* spp.), environmental mastitis pathogens [*E. coli*, non-*aureus* Staphylococci (NAS), *Trueperella pyogenes*, and *S. dysgalactiae*], environmental mastitis pathogen that can also act as contagious mastitis pathogen (*Streptococcus uberis*) (21), fungi (all yeast, mold, and other fungus spp. aggregated), and algae (*Prototheca* spp.) that are frequently isolated from mastitis milk samples and are important causes of bovine mastitis (2, 10–15). The pathogens of genus *Corynebacterium* (*Corynebacterium bovis, Corynebacterium ulcerans*, and all other *Corynebacterium* spp.) were aggregated as *Corynebacterium* spp. and treated as contagious mastitis pathogen as the majority of the isolates reported at the species level included the contagious mastitis pathogen *Corynebacterium bovis*. Likewise, NAS includes all Staphylococci other than *S. aureus*, like *Staphylococcus agnetis, Staphylococcus capitis, Staphylococcus caprae, Staphylococcus chromogenes, Staphylococcus epidermidis, Staphylococcus equorum, Staphylococcus haemolyticus, Staphylococcus hyicus, Staphylococcus sciuri, Staphylococcus simulans, Staphylococcus xylosus*, and those not reported at species level. While most of these pathogens that are grouped as NAS are coagulase negative (formerly grouped as coagulase negative staphylococci), some others are coagulase positive and coagulase variable Staphylococci.

Date of sample submission was categorized by both month and season—Winter (Dec 21–March 19), Spring (March 20–June 19), Summer (June 20–Sep 21), and Autumn/Fall (Sep 22–Dec 20). Year was modeled as a continuous variable. Likewise, the location of the submitting clinic was categorized into Eastern Ontario, Central Ontario, Southwestern Ontario, and Northern Ontario regions (22). The clinics in Toronto was included in Central Ontario. We used R (23) to perform all the statistical analysis. Chi squared goodness-of-fit tests were used to compare between multiple proportions. Univariable logistic regression analysis was performed to determine if the probability of isolating the major mastitis pathogens (outcome) changed with year, month, and/or geography (24). Seasonal relative risk (RR) and the confidence interval (CI) was calculated as described by Brookhart et al. and is interpreted as a RR measure that compares the month with highest incidence to that of the month with the lowest incidence also called Peak-Low Ratio analysis (25). Statistical significance was determined at *P* < 0.05.

## Results

Between 2008 and 2017, a total of 85,979 milk samples were submitted to the AHL for general bacteriological examination resulting in 91,802 test results. The 85,979 samples included 92 composite milk samples.

### Frequency of the Major Mastitis-Associated Pathogens

Between January 1, 2008 and December 31, 2017, the AHL performed milk culture on 85,979 milk samples from dairy cattle in the province of Ontario. The number of samples subjected to milk culture are shown in Table 1. Over the years, the average number of milk samples submitted by a clinic was approximately reduced by half. Consequently, the number of samples tested more than halved from 13,339 to 5,891, resulting in a corresponding decrease in the test results.

**Table 1:** The final data included a total of 85,979 milk samples that were cultured between 2008 and 2017 in the Animal Health Laboratory, Ontario, Canada.

**Year**	**Number of sample submissions**	**Average number of samples per submission**	**Total number of samples tested**	**Total milk culture test results**	**Average number of test results per sample**
2008	1,929	6.91	13,339	13,473	1.01
2009	1,786	5.18	9,249	9,375	1.01
2010	1,711	5.36	9,169	9,281	1.01
2011	1,905	5.40	10,289	10,373	1.01
2012	2,376	4.21	9,992	10,161	1.02
2013	1,964	4.45	8,747	8,943	1.02
2014	1,637	4.05	6,625	7,988	1.21
2015	1,883	3.35	6,312	7,365	1.17
2016	1,866	3.41	6,366	7,459	1.17
2017	1,769	3.33	5,891	7,384	1.25
Total	18,826	4.57	85,979	91,802	1.07

**Figure 1:**
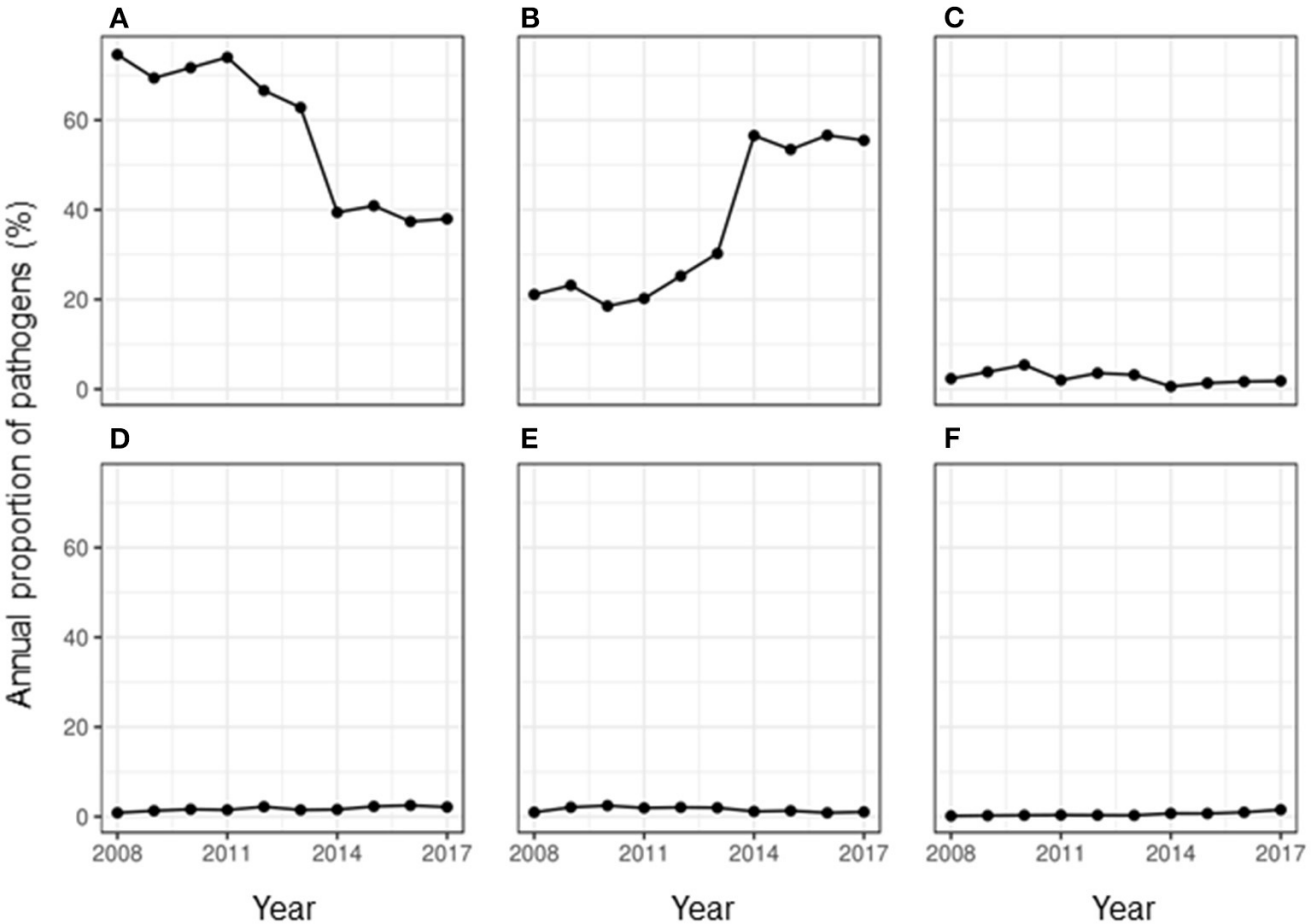
Annual proportion of the major classes of pathogens isolated from milk samples submitted to the University of Guelph, Animal Health Laboratory, Ontario, Canada between 2008 and 2017. **(A)** No growth, **(B)** bacteria, **(C)** contamination, **(D)** algae, **(E)** fungus, and **(F)** mixed growth.

More than half of the milk samples (61.07%, *n* = 85,979) showed no growth of any group of pathogens. The peak season for observing no growth in milk culture was summer with a RR of 1.21 (95% CI: 1.12–1.31) compared to that in winter. During the study period, the proportion of samples that showed no growth almost halved in the year 2017 when compared to the year 2008 (Figure 1). This overall decreasing trend with each year was found to be significant with an odds ratio (OR) of 0.82 (95% CI: 0.81–0.82).

**Figure 2:**
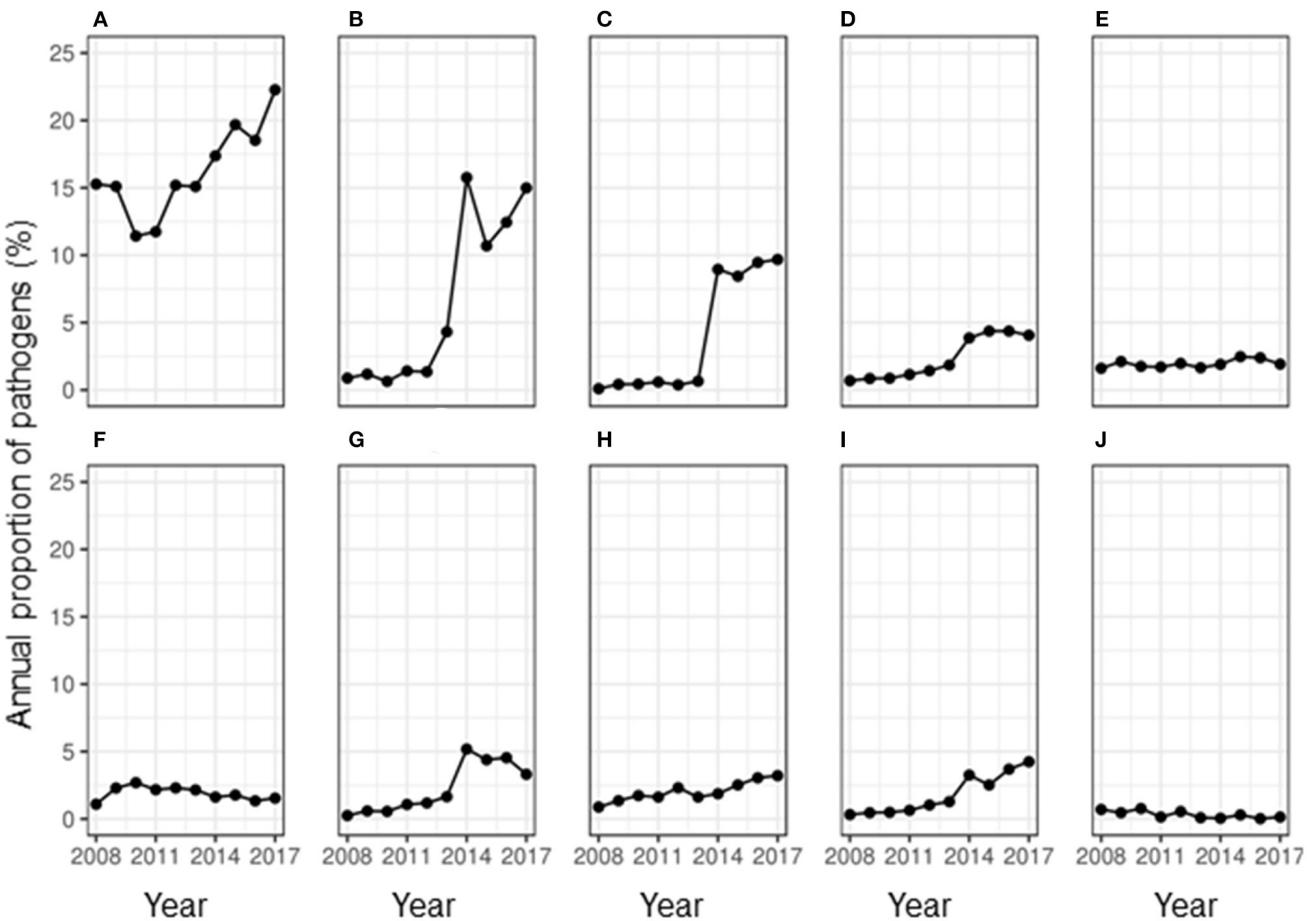
Annual proportion of major mastitis pathogens isolated from 85,988 milk samples between 2008 and 2017 in Ontario, Canada. **(A)** *Staph. aureus*, **(B)** non-aureus Staphylococci, **(C)** Corynebacterium spp., **(D)** *E. coli*, **(E)** *Trueperella pyogenes*, **(F)** Algae, **(G)** *Streptococcus uberis*, **(H)** Fungus, **(I)** *Strep. dysgalactiae*, and **(J)** *Streptococcus agalactiae*.

Less than half of the samples (36.21%, *n* = 85,979) showed any growth, while 2.73% of the samples showed contamination. The overall RR of observing contamination was significantly higher during summer compared to that in winter (RR: 21.69, 95% CI: 2.65–177.68). While the proportion of samples showing contamination fluctuated during the study period, an overall decrease in proportion per year was observed (OR = 0.93, 95% CI: 0.91–0.94) compared to the baseline year of 2008. During the study period, the proportion of isolation of bacteria and algae from a milk sample doubled (Figure 1). Of the uncontaminated milk culture results, 89.60% of the samples (*n* = 31,133) yielded bacterial isolates only, while 1.31% showed mixed cultures of bacteria, fungi and/or algae. Among the major pathogens isolated from the milk samples, most of them were bacterial pathogens (Figure 2) and the top five bacterial species constituted more than two-thirds (78.20%) of the total isolates. Among the fungi, most of the isolates were yeasts (99.56%, *n* = 1,607). Among the *Corynebacterium* spp., 14.02% (359) of the isolates were reported as *Corynebacterium bovis*, three as *Corynebacterium ulcerans* while rest of the isolates were not reported at the species level. Likewise, of the 5.04% of NAS, less than a half of the NAS (44.36%, *n* = 4,337) were reported at species level and of those identified at species level, most of them comprised *S. chromogenes* (69.02%), *S. simulans* (14.45%), *S. epidermidis* (12.99%), and *S. hyicus* (2.13%).

### Temporal Change in Frequency of Isolation of Major Mastitis Pathogens in Milk Samples in Ontario

#### Contagious Mastitis Pathogens

During the study period, there was a fluctuating trend in the proportion of contagious mastitis pathogens isolated from milk. When the number of samples positive for a pathogen was assessed, there was a significant overall increase in the proportion of samples positive for the major contagious mastitis pathogens during the study period (Figure 2). In the year 2008, 15.27% of all the milk samples that were cultured isolated *S. aureus*. While there was a decrease in the proportion of samples isolating *S. aureus* in the year 2010 and 2011, an overall increase in prevalence of *S. aureus* was observed during the study period, such that in 2017, *S. aureus* was isolated from 22.27% of the milk samples. While the prevalence of *Corynebacterium* spp. remarkably increased in the milk samples. during the study period, the positivity rate of *Streptococcus agalactiae* almost halved (Figure 2). Likewise, the prevalence of *S. uberis*, some strains of which are known to act as a contagious pathogen, also remarkably increased in the milk samples. during the study period (Figure 2). The annual odds of increase were 1.06 for *S. aureus* and 1.62 for *Corynebacterium* spp. (Table 2).

**Table 2:** Result of univariable regression analysis of the proportion of samples positive for major mastitis pathogens by year.

**Pathogens**	**Odds ratio**	***P-*value**
	**(95% Confidence interval)**	**(Chi square)**
*Staphylococcus aureus*	1.06 (1.05, 1.07)	<0.01
Non-*aureus* Staphylococci	1.47 (1.45, 1.49)	<0.01
*Corynebacterium* spp.	1.62 (1.59, 1.65)	<0.01
*Escherichia coli*	1.27 (1.25, 1.29)	<0.01
*Trueperella pyogenes*	1.03 (1.01, 1.05)	<0.01
*Streptococcus dysgalactiae*	1.36 (1.33, 1.39)	<0.01
Algae (*Prototheca* spp.)	0.99 (0.97, 1.01)	0.22
Fungi	1.13 (1.11, 1.15)	<0.01
*Streptococcus uberis*	1.33 (1.31, 1.36)	<0.01
Contamination	0.93 (0.91, 0.94)	<0.01
No growth	0.82 (0.81, 0.82)	<0.01

*P, Probability*.

#### Environmental Mastitis Pathogens

Figure 2 show that there was an increase in the proportion of each major environmental pathogen in the milk samples from the year 2008 to 2017. There was a corresponding 17-fold increase in the prevalence of NAS, an environmental pathogen (Figure 2) during the study period. Likewise, the proportion of samples positive for *E*. *coli* increased more than 5-fold from 0.69 to 4.06% and the samples positive for *S. dysgalactiae* increased more than 13-fold during this time. On contrary, the proportion of samples positive for *T. pyogenes* remained similar throughout the study period. The annual odds of increase of prevalence per year were significant for all the environmental mastitis pathogens (Table 2).

#### Minor Mastitis Pathogens

Figure 2 shows that the proportion of samples positive for Algae (*Prototheca* spp.) changed from 1.09% in the year 2008 to 1.54% in the year 2017 without any significant increase or decrease per year from the base year of 2008 (OR = 0.99; 95% CI: 0.97–1.01, *P*-value = 0.22). Likewise, the proportion of samples positive for fungi increased from <1 to 3.21% during the study period with an annual odds of increase of 1.13 (95% CI: 1.11–1.15, *P*-value < 0.01) from the year 2008.

### Seasonality

Tables 3–5 show variation in the monthly frequency of isolation of the mastitis pathogens. The odds of increase or decrease of the prevalence of the pathogens during the months of year compared to that in January showed that there was an increasing odds of the prevalence of pathogens like *S. aureus* during March and April and decreasing odds of isolation during the summer months of August to September (Tables 6–8). Likewise, a higher odds of isolating NAS and *E. coli* from milk was observed in the spring and summer months compared to that in January. In contrast, the odds of isolating *S. dysgalactiae* from a milk sample was lower during summer, although this observation was not statistically significant. While compared to January the odd of isolating fungus was higher for both the summer months (July–September) and autumn months (October and November), the highest odds of isolating algae from a milk sample was observed in winter (February) when compared to that in January.

**Table 3:** Temporal (monthly) and spatial change in the proportion of bovine milk samples positive for various mastitis pathogens between 2008 and 2017 in Ontario, Canada.

**Parameters**		**Percentage of samples showing growth of different mastitis pathogens**			**Total samples per category**
**Category**		***Staphylococcus aureus***	***Corynebacterium* spp**.	***Streptococcus uberis***	
Month	January	7.90	4.32	5.94	6,728
	February	7.85	6.99	7.49	7,001
	March	10.48	7.81	7.93	7,172
	April	9.85	10.76	7.68	6,648
	May	7.78	8.09	0.85	6,461
	June	8.90	7.15	8.42	7,539
	July	10.00	10.80	11.64	9,151
	August	9.11	11.54	12.94	9,595
	September	7.96	7.54	8.79	7,439
	October	7.64	7.62	7.86	7,009
	November	6.73	11.58	7.06	6,073
	December	5.80	5.81	5.39	5,163
Location	Central Ontario	8.35	8.13	8.85	7,165
	Eastern Ontario	28.00	31.92	20.25	22,220
	Northern Ontario	2.93	2.87	2.04	1,952
	Southwestern Ontario	60.72	57.09	68.85	54,642
Total (*N*)		13,411	2,547	1,615	85,979

**Table 4:** Temporal (monthly) and spatial change in the proportion of bovine milk samples positive for various environmental mastitis pathogens between 2008 and 2017 in Ontario, Canada.

**Parameters**		**Number of samples showing growth of different environmental mastitis pathogens (% of total samples)**				**Total samples per category**
**Category**		**Non-*aureus* Staphylococci**	***E. coli***	***Trueperella pyogenes***	***Streptococcus dysgalactiae***	
Month	January	5.42	5.86	8.82	7.47	6,728
	February	5.26	4.06	18.76	5.11	7,001
	March	9.25	5.63	9.12	13.22	7,172
	April	10.72	4.76	9.74	10.23	6,648
	May	7.06	8.53	8.33	9.05	6,461
	June	8.97	10.74	8.21	8.89	7,539
	July	16.16	13.23	8.70	8.42	9,151
	August	13.19	14.39	8.76	9.91	9,595
	September	9.68	11.38	6.80	7.32	7,439
	October	6.11	8.59	7.96	7.00	7,009
	November	4.82	7.78	8.33	7.08	6,073
	December	3.37	5.05	6.49	6.29	5,163
Location	Central Ontario	5.53	2.26	7.90	4.64	7,165
	Eastern Ontario	18.75	18.69	25.05	21.95	22,220
	Northern Ontario	1.38	1.74	2.57	3.54	1,952
	Southwestern Ontario	74.34	77.31	64.48	69.87	54,642
Total (*n*)		4,337	1,723	1,633	1,271	85,979

**Table 5:** Temporal (monthly) and spatial change in the proportion of samples positive for algae and fungi in bovine milk samples between 2008 and 2017 in Ontario, Canada.

**Parameters**	**Category**	**Number of samples showing growth of different minor mastitis pathogens (% of total samples per year)**		**Total samples per category**
		**Algae (*Prototheca* spp.)**	**Fungi (several spp.)**	
Month	January	8.47	5.97	6,728
	February	12.00	6.34	7,001
	March	10.05	7.15	7,172
	April	5.48	6.22	6,648
	May	7.92	5.22	6,461
	June	9.20	7.34	7,539
	July	8.47	12.06	9,151
	August	9.26	13.18	9,595
	September	8.17	12.44	7,439
	October	6.89	10.20	7,009
	November	7.19	7.84	6,073
	December	6.89	6.03	5,163
Location	Central Ontario	9.45	10.07	7,165
	Eastern Ontario	20.72	28.54	22,220
	Northern Ontario	1.89	0.56	1,952
	Southwestern Ontario	67.95	60.82	54,642
Total (*n*)		1,641	1,608	85,979

**Table 6:** Regression analysis of the proportion of samples positive for various mastitis pathogens by month.

**Categories of explanatory variable month**	**Outcome as proportion of samples positive for each of the mastitis pathogens**		
	***Staphylococcus aureus***	**Corynebacterium spp**.	***Streptococcus uberis***

	**Odds ratio (95% confidence interval)**	**Odds ratio (95% confidence interval)**	**Odds ratio (95% confidence interval)**
	**Overall** ***P*****-value** **≤** **0.01**	**Overall** ***P*****-value** **≤** **0.01**	**Overall** ***P*****-value** **≤** **0.01**
January	1.00	1.00	1.00
February	0.95 (0.86, 1.04)	1.57 (1.24, 2.00)^*^	1.21 (0.93, 1.59)
March	1.30 (1.19, 1.42)^*^	1.72 (1.36, 2.18)^*^	1.25 (0.96, 1.64)
April	1.33 (1.21, 1.45)^*^	2.59 (2.07, 3.25)^*^	1.31 (1.00, 1.72)
May	1.03 (0.94, 1.13)	1.98 (1.57, 2.51)^*^	1.56 (1.21, 2.03)^*^
June	1.01 (0.92, 1.10)	1.49 (1.17, 1.90)^*^	1.27 (0.98, 1.66)
July	0.92 (0.84, 1.00)	1.86 (1.5, 2.34)^*^	1.46 (1.14, 1.87)^*^
August	0.78 (0.71, 0.85)^*^	1.9 (1.53, 2.38)^*^	1.54 (1.21, 1.97)^*^
September	0.9 (0.82, 0.98)^*^	1.59 (1.26, 2.03)^*^	1.34 (1.04, 1.75)^*^
October	0.92 (0.83, 1.01)	1.71 (1.36, 2.18)^*^	1.27 (0.98, 1.67)
November	0.93 (0.85, 1.03)	3.07 (2.47, 3.85)^*^	1.32 (1.01, 1.74)^*^
December	0.95 (0.86, 1.05)	1.78 (1.39, 2.28)^*^	1.18 (0.88, 1.59)

* *Represent significant odds ratios*.

**Table 7:** Regression analysis of the proportion of samples positive for environmental mastitis pathogens by month.

**Categories of explanatory variable month**	**Outcome as proportion of samples positive for each of the mastitis pathogens**			
	**Non-*aureus* Staphylococci**	***Escherichia coli***	***Trueperella pyogenes***	***Streptococcus dysgalactiae***

	**Odds ratio (95% confidence interval)**	**Odds ratio (95% confidence interval)**	**Odds ratio (95% confidence interval)**	**Odds ratio (95% confidence interval)**
	**Overall** ***P*****-value** **≤** **0.01**	**Overall** ***P*****-value** **≤** **0.01**	**Overall** ***P*****-value** **≤** **0.01**	**Overall** ***P*****-value** **≤** **0.01**
January	1.00	1.00	1.00	1.00
February	0.93 (0.77, 1.12)	0.66 (0.49, 0.90)^*^	0.95 (0.75, 1.2)	0.65 (0.47, 0.90)^*^
March	1.64 (1.39, 1.93)^*^	0.9 (0.68, 1.19)	0.97 (0.77, 1.22)	1.67 (1.30, 2.17)^*^
April	2.08 (1.77, 2.44)^*^	0.82 (0.61, 1.10)	1.12 (0.89, 1.41)	1.39 (1.07, 1.82)^*^
May	1.37 (1.16, 1.64)^*^	1.53 (1.18, 1.98)^*^	0.98 (0.78, 1.25)	1.27 (0.96, 1.67)
June	1.5 (1.28, 1.78)^*^	1.65 (1.30, 2.11)^*^	0.83 (0.65, 1.05)	1.06 (0.81, 1.40)
July	2.29 (1.97, 2.67)^*^	1.68 (1.33, 2.13)^*^	0.72 (0.57, 0.91)^*^	0.83 (0.63, 1.09)
August	1.75 (1.5, 2.05)^*^	1.74 (1.38, 2.21)^*^	0.69 (0.55, 0.87)^*^	0.93 (0.71, 1.22)
September	1.65 (1.41, 1.95)^*^	1.78 (1.40, 2.27)^*^	0.69 (0.54, 0.89)^*^	0.88 (0.66, 1.18)
October	1.09 (0.91, 1.3)	1.42 (1.10, 1.83)^*^	0.86 (0.68, 1.1)	0.9 (0.67, 1.20)
November	0.98 (0.81, 1.19)	1.48 (1.14, 1.92)^*^	1.05 (0.83, 1.33)	1.05 (0.78, 1.40)
December	0.8 (0.65, 0.99)^*^	1.12 (0.84, 1.50)	0.96 (0.74, 1.23)	1.1 (0.81, 1.48)

* *Represent significant odds ratios*.

**Table 8:** Regression analysis of the proportion of samples positive for mastitis pathogens, no growth, and contamination by month.

**Categories of explanatory variable month**	**Outcome as proportion of samples positive for mastitis pathogens, contamination, or no growth**			
	**Algae (Prototheca spp.)**	**Fungi (several genus)**	**Contamination**	**No growth**

	**Odds ratio (95% confidence interval) overall** ***P*****-value** **≤** **0.01**	**Odds ratio (95% confidence interval) overall** ***P*****-value** **≤** **0.01**	**Odds ratio (95% confidence interval) overall** ***P*****-value** **≤** **0.01**	**Odds ratio (95% confidence interval) overall** ***P*****-value** **≤** **0.01**
January	1.00	1.00	1.00	1.00
February	1.37 (1.10, 1.71)^*^	1.02 (0.77, 1.35)	0.67 (0.50, 0.91)^*^	1.02 (0.95, 1.09)
March	1.12 (0.89, 1.40)	1.13 (0.86, 1.48)	0.79 (0.60, 1.05)	0.75 (0.70, 0.81)^*^
April	0.65 (0.50, 0.85)^*^	1.06 (0.80, 1.40)	0.80 (0.60, 1.06)	0.76 (0.71, 0.82)^*^
May	0.97 (0.76, 1.24)	0.91 (0.68, 1.22)	2.01 (1.59, 2.57)^*^	0.81 (0.75, 0.86)^*^
June	0.97 (0.77, 1.22)	1.10 (0.84, 1.44)	2.96 (2.38, 3.71)^*^	0.72 (0.67, 0.77)^*^
July	0.73 (0.58, 0.93)^*^	1.50 (1.17, 1.92)^*^	3.55 (2.88, 4.41)^*^	0.68 (0.64, 0.73)^*^
August	0.76 (0.60, 0.96)^*^	1.56 (1.23, 2.00)^*^	3.31 (2.69, 4.12)^*^	0.79 (0.74, 0.85)^*^
September	0.87 (0.68, 1.11)	1.91 (1.50, 2.45)^*^	2.83 (2.27, 3.55)^*^	0.80 (0.75, 0.86)^*^
October	0.78 (0.60, 1.00)	1.66 (1.29, 2.14)^*^	1.49 (1.17, 1.92)^*^	0.90 (0.84, 0.97)^*^
November	0.94 (0.73, 1.20)	1.46 (1.12, 1.92)^*^	0.67 (0.49, 0.91)^*^	0.87 (0.81, 0.94)^*^
December	1.06 (0.82, 1.36)	1.32 (0.99, 1.76)	1.14 (0.86, 1.52)	0.93 (0.86, 1.00)

* *Represent significant odds ratios*.

Likewise, Peak-Low Ratio analysis (Table 9) showed that during a year the isolation of most of the pathogens peaked in summer, except for *S. aureus, T. pyogenes*, and *S. dysgalactiae* which peaked in spring.

**Table 9:** Seasonal relative risk of isolation of major mastitis pathogens between 2008 and 2017 in Ontario, Canada.

**Isolates**	**Peak/Low ratio**	**Peak season**
	**(95% confidence interval)**	
*Staphylococcus aureus*	1.36 (1.16, 1.59)	Spring
*Corynebacterium* spp.	1.35 (1, 1.93)	Summer
Non-*aureus* Staphylococci	2.76 (1.96, 3.89)	Summer
*Escherichia coli*	3.04 (1.72, 5.38)	Summer
*Trueperella pyogenes*	1.12 (1, 1.73)	Spring
*Streptococcus dysgalactiae*	1.38 (1, 2.29)	Spring
Fungi	1.08 (1, 1.68)	Spring
Algae (*Prototheca* spp.)	2.11 (1.27, 3.49)	Summer
Contamination	21.69 (2.65, 177.68)	Summer
No growth	1.21 (1.12, 1.31)	Summer

### Spatial Change in Frequency of Isolation of Major Mastitis Pathogens in Milk Samples at Ontario

Tables 3–5 show that frequency of isolation of the major pathogens varied by geographical regions, however, such variation was only significant for some of the pathogens (Table 10). Compared to Central Ontario, in Eastern Ontario, there was a significantly increased odds of samples being positive for *S. aureus*, and *Corynebacterium* spp. while there were decreased odds of a sample being positive for *S. uberis*. In all regions of Ontario, there was an increased odds of isolation of *E. coli*, and *S. dysgalactiae* compared to Central Ontario. In contrast, compared to that of Central Ontario, a significant decreasing odds of isolation of algae and fungi was noted in Eastern Ontario (Table 10).

**Table 10:** Univariable regression analysis of the proportion of samples showing contamination or no growth and those positive for major contagious and environmental mastitis pathogens by location.

**Pathogens**	**Locations**	**Odds ratio (95% confidence interval)**	***P*-value**
*Staphylococcus aureus*	Eastern Ontario	1.10 (1.02, 1.18)^*^	<0.01
	Northern Ontario	1.36 (1.20, 1.54)^*^	
	Southwestern Ontario	0.94 (0.88, 1.01)	
	Central Ontario	1.00	
*Streptococcus uberis*	Eastern Ontario	0.73 (0.60, 0.90)^*^	<0.01
	Northern Ontario	0.84 (0.57, 1.22)	
	Southwestern Ontario	1.02 (0.86, 1.22)	
	Central Ontario	1.00	
*Corynebacterium* spp.	Eastern Ontario	1.28 (1.10, 1.49)^*^	<0.01
	Northern Ontario	1.31 (0.99, 1.71)	
	Southwestern Ontario	0.92 (0.79, 1.07)	
	Central Ontario	1.00	
Non-*aureus* Staphylococcus	Eastern Ontario	1.1 (0.95, 1.27)	<0.01
	Northern Ontario	0.92 (0.68, 1.21)	
	Southwestern Ontario	1.81 (1.59, 2.07)^*^	
	Central Ontario	1.00	
*Escherichia coli*	Eastern Ontario	2.69 (1.95, 3.81)^*^	<0.01
	Northern Ontario	2.85 (1.75, 4.59)^*^	
	Southwestern Ontario	4.57 (3.37, 6.39)^*^	
	Central Ontario	1.00	
*Trueperella pyogenes*	Eastern Ontario	1.02 (0.84, 1.25)	0.64
	Northern Ontario	1.2 (0.83, 1.69)	
	Southwestern Ontario	1.07 (0.89, 1.29)	
	Central Ontario	1.00	
*Streptococcus dysgalactiae*	Eastern Ontario	1.53 (1.16, 2.05)^*^	<0.01
	Northern Ontario	2.84 (1.91, 4.19)^*^	
	Southwestern Ontario	1.99 (1.54, 2.62)^*^	
	Central Ontario	1.00	
*Prototheca* spp. (Algae)	Eastern Ontario	0.70 (0.58, 0.85)^*^	<0.01
	Northern Ontario	0.73 (0.49, 1.06)	
	Southwestern Ontario	0.94 (0.80, 1.12)	
	Central Ontario	1.00	
Fungus	Eastern Ontario	0.91 (0.76, 1.10)	<0.01
	Northern Ontario	0.20 (0.09, 0.37)^*^	
	Southwestern Ontario	0.79 (0.67, 0.94)^*^	
	Central Ontario	1.00	
Contamination	Eastern Ontario	1.09 (0.95, 1.26)	<0.01
	Northern Ontario	0.76 (0.56, 1.02)	
	Southwestern Ontario	0.70 (0.61, 0.81)^*^	
	Central Ontario	1.00	
No growth	Eastern Ontario	0.87 (0.82, 0.92)^*^	<0.01
	Northern Ontario	0.87 (0.78, 0.96)^*^	
	Southwestern Ontario	0.85 (0.81, 0.90)^*^	
	Central Ontario	1.00	

* *Represent significant odds ratios*.

## Discussion and Conclusions

In this retrospective study we have described the frequently isolated pathogens from milk samples in Ontario, Canada and evaluated the major trends for nine major mastitis pathogens. This information will facilitate spatial and temporal comparisons with other studies. The study result is not intended to be a diagnostic guide for veterinarians managing the treatment of bovine mastitis as such decisions should be based on testing of individual samples as far as practically possible. However, information on the most frequently isolated pathogens from milk samples and the general trend in their occurrence will be useful for veterinarians as a reference for decision making in the therapy of mastitis.

The major pathogens responsible for mastitis in Ontario have changed significantly over the years examined in this study. The previously important pathogens have been replaced by pathogens which were considered minor pathogens in the past. While this trend is like that in other countries and other parts in Canada, the findings from this study highlight some differences from other studies. This decreasing trend could be attributed to control efforts toward the causative agent of communicable mastitis. However, control efforts should be continued to reduce not only the major contagious pathogens in Ontario but also environmental, minor, and emerging mastitis pathogens.

While *S. aureus, S. uberis*, and *E. coli* were more frequently isolated from mastitis milk samples in the Atlantic Provinces of Canada between 1994 and 2013 (26), this study shows that in Ontario NAS were more frequently isolated than *E. coli*. In contrast to our study finding, in a Canada wide study *Enterobacteriaceae* (*E. coli* and *Klebsiella* spp.) accounted for more than a quarter of isolates from mastitis milk (10). This study also showed that over the years NAS have increasingly been isolated from the milk samples. At the species level, *S. chromogenes* was the dominant NAS species similar to what was noted in a Canada wide study, the US study and some European studies, however regional variation in the type of NAS species prevalence was noted (27–29). Understanding of the prevalence of NAS at species level is necessary from a disease control and management point of view as infection by some of these species of NAS has been known to be persistent and hence a source of infection to other animals in the herd. Whereas, other species like *S. simulans* are more of an environmental pathogen (29, 30).

This study shows the low prevalence of some important emerging mastitis pathogens. There was a consistent presence of a potential pathogen *Aerococcus* spp. in Ontario milk samples since 2014. While no confirmatory evidence is available, *Aerococcus viridans* has been implicated in subclinical bovine mastitis (31). Likewise, in this study at least 1% of the isolates were identified as *Prototheca* spp., an algae responsible for incurable mastitis. *Prototheca* spp. is an emerging pathogen in Canada with it not been reported between 2003 and 2005 (6). A case-control study in 2011 however determined mean within-herd prevalence of *Prototheca* spp of 5.1% (32). The present study also reported a low prevalence of an important contagious mastitis pathogen *Mycoplasma* in milk. In a Canada wide study conducted between 2003 and 2005, no *Mycoplasma* was detected in milk samples from clinically infected animals (6). A low prevalence of *Mycoplasma* in bulk milk samples was reported in a study from Prince Edward Island, Canada (33). In the current study, no *Mycoplasma* was detected in milk samples from 2008 to 2012, however, between 2012 and 2018 a total of 98 isolates were identified. While Canada has historically maintained a low prevalence of *Mycoplasma* compared to other countries (34), it may represent an emerging concern to be monitored.

This study reports interesting seasonal trends of isolation of major mastitis pathogen and clustering of these pathogens by the location of the clinic submitting the samples indicating the possible necessity of seasonal and regional mastitis control approaches. However, caution should be taken while interpreting this as the clustering could be due to stochastic effect of sample submission by the clinics in the geography as some of the clinics conduct milk culture themselves and some sample submission could be to identify the pathogens that they are not capable of doing at their laboratories. In contrast to our study, a Danish study reported that the incidence rate of *S. aureus, E. coli* (pastured herd), and *S. dysgalactiae* clinical mastitis was found to be the highest in the winter months (35). Dairy herds in Ontario are predominantly of tie stall types (36) which could be one reason for this unique observation as it was found in a previous study that in case of confined herds, incidence rate of *E. coli* clinical mastitis was higher in summer than in winter (35). The frequency of isolation of *S. uberis* was higher not only in summer, similar to what was found in the Danish study where the highest incidence rate of *S. uberis* clinical mastitis was observed in summer (August), which was associated with pasture (35), but also in autumn.

The spatio-temporal change in the test results could also be a result of a decrease in sample submissions over the years possibly as a result of greater use of in-clinic identification techniques, a decrease in farms and/or animals over the years (37), improvement in the culture techniques, adoption of improved sample collection and transportation, and implementation of mastitis control measures on farm.

This study is based on passively collected data which have limitations. It is a non-systematic study and since laboratory submission requires a veterinarian, the decision to submit a sample will be influenced by the farmer. Likewise, information on farm type, age of animal, their lactational status, clinical form of the disease, and/or treatment history is not known. The availability of this information would have improved our understanding of seasonal and regional variations that were observed in this study. Additionally, some of the submissions may have been a result of a research study on mastitis or on-farm surveillance. However, information was not available to identify such submissions. In this study, we have considered that all the samples were from cattle in either clinical or sub-clinical stages of mastitis. A subset of samples may have originated from animals with a history of treatment failure. Likewise, the samples could be subject to selection bias and likely underrepresent milk samples from mastitis cattle in Ontario as many clinics and farms have their own regular mastitis testing programs in place. Likewise, some findings can also be attributed to the improvement in diagnostic techniques at the AHL as since 2011 spectrometric techniques are being used to identify bacterial species. While the use of an in-house clinic lab can be attractive for improved timeliness, AHL is one of the major laboratories in Ontario, and Ontario laboratory samples are subsidized making submission more economically attractive. Therefore, the milk samples are expected to be representative of mastitis milk samples in Ontario. In addition, the representativeness of these samples could also have been affected by farm economics and disease outbreaks as they have been shown to influence laboratory sample submissions (38).

It is a valid expectation that the culture of milk originating from a mastitis cow would focus on all the pathogens that are known to cause mastitis. Likewise, the pathogens that were isolated and hence reported might come from the milk, or the environment of the animal (skin, farm, milking machine, human handlers, etc.). Nevertheless, knowledge of this is important as it is known that some of the environmental pathogens can persist in the farm environment and be a constant source of infection for the animals. Despite all these limitations, there is a value of the information obtained from these passive surveillance data.

Bulk milk samples are used to estimate the prevalence of mastitis pathogens in a region (33, 39). However, the information they provide can be of limited value as the probability of detecting a pathogen will be reduced many fold by the dilution effect of the bulk milk and the inherent limit of detection of culture techniques especially if only a small fraction of animals are infected in a herd. Testing milk samples from individual quarters is valuable but is more costly making prevalence studies challenging. In this context, information on the prevalence of mastitis pathogens can be obtained using passive surveillance data generated by a diagnostic laboratory, as has been attempted in this study.

A significant number of samples had a culture negative test result, and this reduced significantly during the study period. Reduction in no growth is possibly due to the improvement in sampling of milk and culture techniques. However, it is also possible that the clinics submitted the higher proportion of culture positive samples to the AHL for confirmation. Likewise, the diversification of culturing techniques would have enabled the capacity to identify the presence of pathogens like *Mycoplasma* which require special media. Given the low prevalence of *Mycoplasma* in Canada and particularly Ontario, this however looks less likely. Often culture negative test results are attributed to infection by pathogens which are short lived such as *E. coli* and pathogens which are shed in a cyclical manner such as *S. aureus* if a single sample has been tested (40, 41). The simultaneous reduction of the proportion of samples positive for *E. coli* and *S. aureus* could explain the reduction in culture negative test results. Likewise, as previously mentioned, some of the data in this study may come from research studies like a case-control study conducted in 2011, which might have overestimated the culture negative test result (32).

The present study confirms that the prevalence of mastitis pathogens that do not respond to antibiotic treatment is substantial in the milk samples obtained from Ontario cattle between 2008 and 2017 similar to what was observed Canada wide, in the United States, and in the European countries (2, 3, 42, 43). Likewise, we observed that a large proportion of the mastitis milk was negative on culture as reported previously (24). Thus, the use of antibiotics in most instances of mastitis without the identification of the pathogen and their antimicrobial sensitivity result can be unnecessary. The knowledge of the major pathogens prevalent in Ontario farms will aid veterinarians in evaluating their mastitis treatment protocols and management decision making thereby reducing the unnecessary use of antibiotics, as mastitis is considered one of the main reasons for antibiotic use on a dairy farm (44, 45), and hence improving the future prognosis of mastitis treatment. Similarly, this approach can be adopted in other geographies for estimating the prevalence of major mastitis pathogens by utilizing the passive surveillance data originating from diagnostic laboratories.

## Data Availability Statement

The data analyzed in this study is subject to the following licenses/restrictions: On request, the de-identified dataset used in this study can be made available for solely scholarly purposes. Requests to access these datasets should be directed to agreer@uoguelph.ca.

## Author Contributions

KA, GB, DS, and AG contributed to the concept of the study and data analysis. KA conducted the data analysis and wrote the initial draft of the manuscript. All the authors contributed to and approved the final manuscript.

## Funding

This work was supported by funding from the Canadian Institutes of Health Research (CIHR), under the European Commission's Joint Programming Initiative on Antimicrobial Resistance (5th Joint Call).

## Conflict of Interest

The authors declare that the research was conducted in the absence of any commercial or financial relationships that could be construed as a potential conflict of interest.

## Publisher's Note

All claims expressed in this article are solely those of the authors and do not necessarily represent those of their affiliated organizations, or those of the publisher, the editors and the reviewers. Any product that may be evaluated in this article, or claim that may be made by its manufacturer, is not guaranteed or endorsed by the publisher.
